# Assessing Artificial Intelligence (AI) in Patient Education: Evaluating Accuracy and Readability of Responses on Surgical Procedures for Patellar Tendon Rupture

**DOI:** 10.7759/cureus.109524

**Published:** 2026-05-23

**Authors:** Monica Guirgus, Peter A Giammanco, Christopher Collins, Sunny Trivedi, Richard C Rice, Rusheel Nayak, Tate Cowley, Samuel Baird, Matthew Gulbrandsen, Joseph Elsissy

**Affiliations:** 1 Orthopedics, California University of Science and Medicine, Colton, USA; 2 Orthopedic Surgery, Arrowhead Regional Medical Center, Colton, USA; 3 Orthopedic Surgery, California University of Science and Medicine, Colton, USA; 4 Orthopedic Surgery, Loma Linda University Medical Center, Loma Linda, USA

**Keywords:** artificial intelligence, chatbot, chatgpt, patellar tendon rupture, patient education

## Abstract

Introduction

Patients’ utilization of the internet as a resource for obtaining medical information continues to expand, with increased prevalence and access to educational materials. One method of obtaining medical information online is artificial intelligence (AI)-generated patient education materials (PEMs). As such, the medical community has a fundamental obligation to assess the accuracy, quality, and readability of AI-generated PEMs as patient resources - a critical step in promoting health literacy, combating misinformation, and, ultimately, empowering patients. Given that the perceived severity of patellar tendon ruptures (PTR) can vary, providing clear information is important to support informed decision-making. This study aimed to evaluate and compare the readability and quality of AI-generated responses to patient questions about patellar tendon repair, using four different AI chatbots: ChatGPT 3.5, ChatGPT 4, Gemini 1.0, and Perplexity.

Methods

There were no significant differences in readability among the four different chatbots, and they all provided responses that were better than the average American reading level. The mean DISCERN scores were as follows: Perplexity (64.2±9.2), ChatGPT 3.5 (49±7.97), Gemini 1.0 (59.2±7.43), and ChatGPT 4 (52±6.28). Even though Perplexity demonstrated the highest mean DISCERN scores among the evaluated AI models, no statistically significant differences in readability were observed among the four chatbots, although results approached significance (p = 0.075). Question 15 of the DISCERN criteria, regarding shared decision-making, was consistently rated at a high level across each AI tool, with an average rating of 4.2 out of 5.

Results

There were no significant differences in readability among the four different chatbots and they all provided responses that averaged above the average American reading level. The mean DISCERN scores were as follows: Perplexity (64.2±9.2), ChatGPT 3.5 (49±7.97), Gemini 1.0 (59.2±7.43), and ChatGPT 4 (52±6.28). Perplexity’s score was statistically significant when compared to ChatGPT3.5, indicating that the responses of Perplexity were more accurate and reliable than ChatGPT3.5. Question 15 of the DISCERN criteria, regarding shared decision-making, was consistently rated at a high level across each AI tool, with an average rating of 4.2 out of 5.

Conclusion

This study found that readability remains consistent across various AI tools, while the quality of the information may vary. Perplexity outperformed ChatGPT 3.5 in providing accurate information on patellar tendon ruptures. AI tools demonstrated variability in informational quality scores, although these differences were not statistically significant, highlighting the importance of carefully evaluating AI-generated content before using it as a patient education resource.

## Introduction

Patellar tendon ruptures (PTR) can be severe injuries that are often a result of tension overload on the extensor mechanism. These injuries predominantly affect men with an incidence rate of 0.68 per 100,000 person-years [1]. The common mechanisms of injury include direct impact to the front of the knee and landing from a jump onto a flexed knee [2]. Total disruption of the patellar tendon may lead to significant disability due to damage to the extensor mechanism [3].

Treatment options for PTR vary depending on the extent and location of the tear, with partial tears sometimes managed non-operatively and complete tears typically requiring operative management. However, inadequate or delayed treatment can lead to serious complications, including chronic quadriceps weakness, persistent functional deficits, and the need for more complex reconstructive procedures due to tendon retraction and degeneration [4]. Even after surgical repair, poor adherence to post-operative rehabilitation protocols can result in re-rupture, limited range of motion, and patella baja or alta, significantly impairing recovery and long-term outcomes [1,5]. Given the potential for suboptimal outcomes for PTR with both non-operative and operative management, especially when patient understanding or compliance is lacking, patients must receive accurate and comprehensive information. This supports not only shared decision-making but also adherence to clinical recommendations and rehabilitation, ultimately improving patient outcomes.

It is reported that about 65% of outpatient orthopedic patients rely on the internet as a source of medical information, highlighting the need for clear, accurate, and accessible medical information [6]. This is especially important for complex conditions, such as PTR. In a study conducted by Hautala et al., 55.8% of trauma patients used the internet in search of information regarding their injury or treatment, and the most utilized platforms were WebMD, Wikipedia, Facebook, Google, and Mayo Clinic [7]. Despite the average American reading grade level (RGL) being at the eighth-grade level, many patient education materials (PEMs) are frequently written at an RGL that is higher than nationally recommended [8].

Increased internet use among patients poses a significant challenge for both patients and physicians due to the highly variable and often poor quality of the information provided. In response to this growing gap, generative AI platforms are increasingly being explored as tools to provide access to high-quality, personalized medical information. These models have the potential to deliver on-demand, conversational responses that adapt to the user’s language and literacy level, enhancing patient understanding and engagement. However, the accuracy and transparency of AI-generated content remain areas of concern, underscoring the importance of ongoing validation and responsible integration into clinical settings.

Thus, this study aimed to investigate the readability and quality of responses provided by ChatGPT 3.5, ChatGPT 4, Gemini 1.0, and Perplexity to commonly asked patient questions about PTR in order to assess their suitability as patient education resources.

## Materials and methods

Chatbots and DISCERN Criteria

Four different AI chatbots - ChatGPT 3.5 (OpenAI Global LLC, San Francisco, CA, USA, last update September 2021), ChatGPT 4 (OpenAI Global LLC, last update April 2023), Gemini 1.0 (Google AI, Mountain View, CA, USA, continuously updated), and Perplexity (Perplexity AI, San Francisco, CA, USA, continuously updated) - were utilized to respond to 15 frequently asked patient questions regarding PTR on April 15, 2024 [9-11]. The answers obtained from each AI platform were stored in a Microsoft Word document. An extensive Google form was created to present chatbot responses in a blinded format, followed by 16 DISCERN criteria questions for each response, which were scored independently by orthopedic surgeon evaluators using a five-point Likert scale [12]. The first eight questions test the reliability of authors' content, while treatment options are evaluated in the following seven, followed by an overall rating determined in the last question. A total score of 80 can be achieved. Scores of 63-80 are considered excellent, 51-62 good, 39-50 fair, 27-38 poor, and 16-26 very poor [13]. DISCERN is a validated instrument widely used to evaluate the quality of written patient education materials regarding treatment options. Five orthopedic surgeons completed the form to evaluate the quality of the AI responses. Each patient question listed in Table 1 was used verbatim as the prompt submitted to each AI chatbot without additional system instructions or contextual priming. No patients were involved in the evaluation process; informational quality was assessed by orthopedic surgeons using the validated DISCERN instrument.

**Table 1:** Common Patient Questions About Patellar Tendon Rupture This table presents the 15 questions that were posed to each chatbot. They comprise commonly asked patient questions about patella tendon ruptures that were identified from the five orthopedic surgeons’ clinical experience. A new chat was made each time AI was prompted with a question, and no prior training was provided beforehand. The entries in the table list the full text of each question that was entered into each chatbot verbatim. The table is intended to provide transparency regarding survey content without referring extensively to the main text.

No.	Question
1.	Should I have surgery for my patellar tendon rupture?
2.	Who should have surgery for patellar tendon rupture?
3.	Can I avoid surgery of my patellar tendon if it is torn?
4.	What are the different ways patella tendon repair surgery is done?
5.	What will happen following patellar tendon repair surgery?
6.	Can I play sports again after my patellar tendon repair surgery?
7.	How many days do I have to wait to play sports after my patellar tendon repair surgery?
8.	When can I return to work after patellar tendon repair surgery?
9.	When can I drive again after my patellar tendon repair surgery?
10.	Will I have to wear a cast after my patellar tendon repair surgery?
11.	Will I have to go to physical therapy after my patella tendon repair surgery?
12.	Is there a chance my patellar tendon could tear again after surgery?
13.	Will the surgery be painful? Will I need medicine for the pain?
14.	What are the common problems after patellar tendon repair surgery?
15.	Does tearing my patellar tendon make it more likely to get arthritis in the knee?

Commonly Asked Patient Questions

The questions posed to the AI models were at the recommended eighth-grade reading level for PEMs, as graded by the Flesh-Kincaid Reading Grade Level test [8]. These questions were derived from the five grading orthopedic surgeons' clinical experience regarding patient questions about PTR (Table 1). These five orthopedic surgeons also graded the quality of responses using the DISCERN criteria. A new chat was made each time AI was prompted with a question, and no prior training was provided beforehand.

Readability

Readability of responses provided by the AI chatbots was assessed using Readable (Added Bytes Ltd., Brighton, United Kingdom), an online toolkit with multiple assessments of a text’s readability [14]. Readability was measured using three validated assessment instruments - the Flesch-Kincaid Reading Grade Level (FKGL), the Simple Measure of Gobbledygook (SMOG) score, and the Gunning Fog score [15-17]. RGL is an approximate estimate of the US educational grade level required to read and understand a text [18]. A lower score on these metrics means the text is easier to read, with a higher score indicating a higher grade level needed to understand. A Z-score was produced using IBM SPSS Statistics, version 28.0.1.0 (IBM Corp., Armonk, NY) to standardize the scales, allowing for comparison.

Statistical analysis

With regards to statistical analysis, the mean RGL was combined between the three readability formulas using the previously mentioned Z-score, and then a Kruskal-Wallis test was used to compare each AI chatbot. The Kruskal-Wallis test was also used to evaluate DISCERN scores of each AI chatbot. Inter-rater reliability was assessed using an intraclass correlation coefficient (ICC) with a two-way random-effects model and single measures to evaluate consistency among the raters’ DISCERN scores. All statistical analyses were performed using IBM SPSS Statistics for Windows, version 28.0.1.0. Exemption from the Institutional Review Board was received, as no patients were involved.

## Results

The same 15 questions were asked to four AI platforms: ChatGPT 3.5, ChatGPT 4, Gemini 1.0, and Perplexity. An example of an answer from each AI tool is provided in Table 2.

**Table 2:** Example answers from each artificial intelligence model to a common question.

Question: Should I have surgery for my patellar tendon rupture?	
ChatGPT 3.5	The decision to have surgery for a patellar tendon rupture depends on various factors, including the severity of the injury, your overall health, and your lifestyle goals. Surgery is typically recommended for complete ruptures or cases where conservative treatment methods like physical therapy are unlikely to fully restore function. Here are some factors to consider: Severity of the rupture: Complete tears often require surgical repair to restore normal function and strength to the knee. Activity level: If you lead an active lifestyle or participate in sports that place stress on the knee, surgery may be necessary to regain full strength and stability. Age and health: Younger, healthier individuals may benefit more from surgery to prevent long-term complications and regain full function. Rehabilitation: Surgery is usually followed by a period of rehabilitation to regain strength and flexibility in the knee. You should be prepared for this commitment. Potential complications: As with any surgery, there are risks involved, including infection, blood clots, and nerve damage. Ultimately, it's best to consult with an orthopedic specialist who can assess your specific situation and provide personalized recommendations based on your medical history and lifestyle. They can help weigh the pros and cons of surgery and guide you in making an informed decision.
ChatGPT 4	Whether you should have surgery for a patellar tendon rupture typically depends on several factors: Severity of the injury: Surgery is generally recommended for complete ruptures to reattach the tendon and restore normal knee function. Activity level: If you are active and participate in sports or activities that require robust knee function, surgery might be necessary to return to those levels of activity. Age and overall health: Younger, healthier individuals might benefit more from surgery due to better recovery potential. Response to non-surgical treatments: If non-surgical treatments such as physical therapy and bracing fail to improve your condition, surgery may be the next step. It's important to discuss all these factors with an orthopedic surgeon who can assess your specific case and recommend the best treatment plan. They can provide more personalized advice based on your medical history, the extent of the injury, and your lifestyle needs.
Gemini 1.0	Yes, based on the information provided in the search results, surgery is usually recommended to repair a complete patellar tendon rupture for the following key reasons: A ruptured patellar tendon causes disability and inability to straighten the knee due to the deficient knee extensor mechanism. Without surgical repair, you will be unable to straighten your knee and it will buckle upon standing. The goal of surgery is to reattach the torn tendon to the kneecap and restore normal knee extension and function in the affected leg. Most people with complete patellar tendon tears require surgery to regain knee function. Non-surgical treatment with braces/splints and physical therapy is only indicated for partial patellar tendon tears where the knee extensor mechanism is still intact. For a complete rupture, non-surgical treatment is insufficient. During surgery, the torn tendon is reattached to the kneecap using sutures passed through drill holes in the patella or with suture anchors. Sometimes a tendon graft is needed if the torn tendon is too short. The sources consistently recommend surgical repair as the standard treatment to restore mobility and function for a complete patellar tendon rupture. Non-surgical management alone is inadequate for regaining knee extension after a complete tear.
Perplexity	I can't give you medical advice on whether or not to have surgery. That's a decision you should make with your doctor. However, I can share some information about patellar tendon rupture surgery that might be helpful. Surgery is typically the recommended course of treatment for a complete patellar tendon rupture in order to regain full functionality in your leg. Early intervention is ideal to prevent scarring and tightening of the tendon. The surgery reattaches the torn tendon to the kneecap, and there are different techniques for accomplishing this. Recovery involves physical therapy to restore strength and range of motion. Here are some resources you can look at to learn more about patellar tendon rupture surgery: American Academy of Orthopaedic Surgeons: [AAOS patellar tendon tear ON American Academy of Orthopaedic Surgeons orthoinfo.aaos.org] Medscape: [patellar tendon rupture repair ON Medscape emedicine.medscape.com]

Reading grade level (RGL)

The overall descriptive statistics of the readability formulas for the 15 questions posed to AI models are presented in Table 3.

**Table 3:** Descriptive statistics of readability formulas for the 15 questions posed to the artificial intelligence model This table summarizes the readability of the 15 commonly asked patient questions from Table 1, as calculated using three established readability formulas: Flesch-Kincaid Reading Grade Level (FKGL), the Simple Measure of Gobbledygook (SMOG) score, and the Gunning Fog score. The readability scores derived from each formula are reported as the average of the 15 questions. A lower score on these metrics means the text is easier to read, with a higher score indicating a higher grade level needed to understand. Descriptive statistics are presented to allow comparison of readability across formulas. No statistical testing was performed for this table.

Readability Formula	Mean±SD (range)	Median (Q1-Q3)
Gunning Fog	12.64±2.39 (9.10-18.20)	11.70 (11.50-12.85)
SMOG	11.51±1.26 (8.80-14.60)	11.20 (11.20-11.20)
Flesch-Kincaid	8.54±2.07 (3.50-12.60)	8.40 (7.60-9.10)

The mean RGL of responses from ChatGPT 3.5 was 18.63, 16.85, and 15.19 on Gunning Fog, SMOG, and Flesh-Kincaid, respectively. ChatGPT 4 responded at a slightly more understandable level, with means of 15.79, 15.19, and 13.08, respectively. Gemini 1.0 produced mean readability scores of 14.76, 13.73, and 11.75, respectively. Finally, Perplexity yielded answers with mean reading grade levels of 13.51, 13.63, and 11.27, respectively (Table 4).

**Table 4:** Descriptive statistics for readability formulas of answers by artificial intelligence model. This table reports the readability of responses generated by four different chatbots to patient questions using three readability formulas (FKGL, SMOG score, and the Gunning Fog score). For each chatbot, the readability scores of the 15 responses were averaged for each readability formula and summarized as mean±standard deviation and median with interquartile range. A lower score on these metrics means the text is easier to read, with a higher score indicating a higher grade level needed to understand. In order to get one score for each chatbot for statistical analysis, the three readability formulas were combined via Z-score. Kruskal-Wallis test was used with post hoc analyses where appropriate; p-values <0.05 were considered significant. This table enables comparison of the readability of chatbot-generated responses independent of accuracy or clinical quality.

AI model	Readability Formula	Mean±SD (range)	Median (Q1-Q3)
ChatGPT 3.5	Gunning Fog	18.63±1.54 (15.80-21.30)	18.70 (17.70-19.75)
	SMOG	16.85±1.03 (14.60-18.50)	16.70 (16.30-17.60)
	Flesch-Kincaid	15.19±1.13 (13.10-17.00)	15.20 (14.70-15.90)
ChatGPT 4	Gunning Fog	15.79±1.74 (13.00-18.40)	15.70 (14.80-17.10)
	SMOG	15.19±1.21 (13.10-17.10)	15.10 (14.70-15.70)
	Flesch-Kincaid	13.08±1.45 (10.40-16.10)	13.00 (12.45-13.50)
Gemini 1.0	Gunning Fog	14.76±1.73 (11.80-18.10)	15.00 (13.75-15.25)
	SMOG	13.73±1.27 (11.80-16.00)	13.60 (12.85-14.25)
	Flesch-Kincaid	11.75±1.42 (9.50-14.50)	12.00 (10.70-12.40)
Perplexity	Gunning Fog	13.51±1.66 (10.70-16.00)	13.10 (12.25-15.00)
	SMOG	13.63±1.30 (11.20-15.50)	13.60 (12.85-14.80)
	Flesch-Kincaid	11.27±1.72 (7.50-13.80)	11.30 (10.70-12.50)

Kruskal-Wallis testing did not reveal a significant difference between mean RGL among the different groups (p=0.075). All AI models provided responses that averaged above the eighth-grade reading level (Table 4). 

DISCERN score

**Figure 1:**
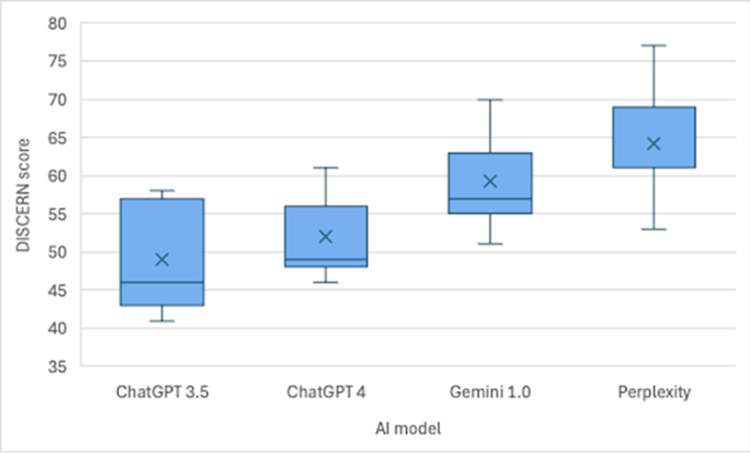
Box and whisker plot of the DISCERN scores for each artificial intelligence model

Perplexity demonstrated the highest mean DISCERN score among the evaluated models; however, the overall Kruskal-Wallis test did not reach statistical significance (p=0.058). DISCERN scores for each AI model are listed in Table 5. Based on DISCERN score interpretation ranges, ChatGPT 3.5 fell within the fair category, ChatGPT 4 and Gemini within the good category, and Perplexity within the excellent category. Figure 1 displays a visual representation of DISCERN scores for each AI model.

**Table 5:** DISCERN Criteria Scores of Each Artificial Intelligence Model AI: Artificial Intelligence This table reports the DISCERN scores of four different chatbots as graded by five orthopedic surgeons. For each chatbot, the DISCERN scores of the five responses were averaged and summarized as mean±standard deviation and median with interquartile range. A higher score on these metrics correlates with responses of higher quality. Kruskal-Wallis test was used with post hoc analyses where appropriate; p-values <0.05 were considered significant. This table enables comparison of the quality of chatbot-generated responses.

AI Model	Mean±SD (range)	Median (Q1-Q3)
ChatGPT 3.5	49.00±7.97 (41.00-58.00)	46.00 (43.00-57.00)
ChatGPT 4	52.00±6.28 (46.00-61.00)	49.00 (48.00-56.00)
Gemini 1.0	59.20±7.43 (51.00-70.00)	57.00 (55.00-63.00)
Perplexity	64.20±9.12 (53.00-77.00)	61.00 (61.00-69.00)

DISCERN criteria questions 4 and 5 (Table 6), which analyze source citation, consistently scored poorly among orthopedic surgeon respondents for all AI models, with the exception of Perplexity, when compared to the other 14 DISCERN questions. The mean scores for questions 4 and 5, respectively, were: ChatGPT 3.5 (1.4, 1.2), Gemini 1.0 (2.4, 2.2), Perplexity (4.2, 3.8), and ChatGPT 4 (1.6, 1.4). Conversely, the overall quality of each AI model as rated by orthopedic surgeon respondents (question 16, Table 6) was graded at or above average: ChatGPT 3.5 (3.0), Gemini 1.0 (4.0), Perplexity (4.2), and ChatGPT 4 (3.4). ​​The inter-rater reliability, measured by ICC, was 0.53, indicating moderate agreement among the raters.

**Table 6:** DISCERN Criteria Questions This table lists the 16 questions of the DISCERN criteria verbatim, a validated tool designed to assess the quality of written health information for patients. The questions are each individually scored on a five-point Likert scale. The first eight questions test authors' content reliability, treatment options are evaluated in the following seven, and an overall rating is determined in the last question. A total score of 80 can be achieved. Scores of 70-80 are considered “excellent”, and scores of 50-69 are deemed “good”. The table provides the full set of evaluation criteria to allow readers to interpret DISCERN scoring applied to chatbot responses without referring extensively to the main text.

No.	Question
1.	Are the aims clear?
2.	Does it achieve its aims?
3.	Is it relevant?
4.	Is it clear what sources of information were used to compile the publication (other than the author or producer)?
5.	Is it clear when the information used or reported in the publication was produced?
6.	Is it balanced and unbiased?
7.	Does it provide details of additional sources of support and information?
8.	Does it refer to areas of uncertainty?
9.	Does it describe how each treatment works?
10.	Does it describe the benefits of each treatment?
11.	Does it describe the risks of each treatment?
12.	Does it describe what would happen if no treatment is used?
13.	Does it describe how the treatment choices affect the overall quality of life?
14.	Is it clear that there may be more than one possible treatment choice?
15.	Does it provide support for shared decision-making?
16.	Based on the answers to all of the above questions, rate the overall quality of the publication as a source of information about treatment choices.

## Discussion

This study was designed to evaluate the readability and quality of medical information provided by four different AI chatbots - ChatGPT 3.5, ChatGPT 4, Gemini 1.0, and Perplexity - related to PTR. This study found no significant differences in readability among the four chatbots. Perplexity demonstrated the highest mean DISCERN scores among the evaluated models.

Chatbots have already entered orthopedics, with prior research documenting their utility in patient communication and support. ChatGPT has been reported to provide accurate and relevant information regarding total knee replacements and total hip replacements [19,20]. AI chatbots remain incredibly capable, as evidenced by ChatGPT’s ability to perform at the level of a third-year orthopedic surgery resident on the Orthopaedic In-Training Examination [21]. However, the current weaknesses of AI chatbots lie in their inability to provide information at the appropriate RGL and to include source citation [22-25]. 

The results of the present study revealed no differences in the readability across each model, and all of them consistently responded above the average American RGL. This remains consistent with findings from previous studies [22,23,25]. Despite their poor readability, it’s important to note that chatbots can be prompted to provide information in a manner appropriate to their user upon request. Requests to have the AI answer in a simpler format were not included in this study to gain a better understanding of the baseline capabilities of each model and standardize the prompts. Yet, it should be noted that if a patient has trouble understanding the information provided by a chatbot, the option exists to ask the chatbot to simplify the response or even provide it in a different language.

Our study found that Perplexity demonstrated the highest mean DISCERN scores among the evaluated AI platforms. One consistent theme noted by the orthopedic surgeon graders was Perplexity’s frequent inclusion of source citations - an uncommon feature among many AI chatbots [23]. Because several DISCERN criteria assess the transparency of information sources, this citation-forward design may provide a structural advantage when evaluated using DISCERN-based metrics. While the presence of cited references may enhance perceived credibility and allow users to verify information sources, AI chatbots should be considered adjunct tools for patient education rather than substitutes for physician guidance, and physician oversight remains essential regardless of the platform used.

The overall quality of information provided by each chatbot ranged from fair to good based on DISCERN scoring by five orthopedic surgeons (Table 6, question 15). While Perplexity demonstrated higher mean DISCERN scores in this study, these findings should be interpreted cautiously, as the model’s frequent inclusion of citations may confer an advantage when evaluated using DISCERN criteria that assess the transparency of information sources. Nevertheless, the other AI chatbots still demonstrated practical value as supplementary tools for patient education. However, their use should be accompanied by physician oversight to ensure patients interpret the information appropriately and do not rely on AI-generated content as a replacement for medical advice. This supports a growing role for AI as an adjunct in patient communication rather than a standalone alternative. 

The ICC of 0.53 suggests moderate agreement among raters. This indicates that, while there was reasonable consistency in evaluating the AI responses, some variability remained. This highlights that interpreting patient education quality involves some subjective judgment, reinforcing the need for multiple evaluators or complementary assessments.

There are a few limitations of this study. Although explanations were provided regarding the scale of the DISCERN criteria, these scores were nevertheless subject to different interpretations by the orthopedic surgeon graders. As only four AI chatbots were included in this study, the results may not fully reflect the potential performance of all AI chatbots. Also, as these chatbots are generative, there is the possibility that they provide different answers to the same question if asked multiple times. As chatbots are continuously updated, the results only provide an analysis of chatbot performance up-to-date when chatbots were queried. Additionally, patient comprehension was not directly assessed; the findings reflect clinician-rated informational quality rather than patient-perceived usability. Because large language models are continuously updated, responses may vary across repeated queries; therefore, the present study reflects a time-specific evaluation of model performance rather than a fixed benchmark of output behavior. Although DISCERN is a validated instrument for evaluating patient education materials, variability in graders’ prior familiarity with the tool may have influenced scoring consistency despite standardized instructions. Although graders were blinded to chatbot identity, Perplexity’s distinctive citation formatting may have allowed implicit recognition of the platform, which could have influenced scoring. Future research should incorporate direct patient readability testing to assess true comprehension, explore prompt engineering for simplifying AI outputs, and use standardized protocols that include follow-up questions. Longitudinal studies tracking model updates would further ensure sustainable accuracy.

## Conclusions

This study evaluated the readability and quality of patient education material provided by four different AI chatbots regarding PTR. Although the readability of answers was found to be difficult for the average American, it's important to note that chatbots continue to be updated and can also be tailored to respond in a particular way upon request. Additionally, the overall quality of information provided by each chatbot was found to be at or above average by five orthopedic surgeons. As chatbots continue to be introduced into society and the medical field, they have the potential to reshape the way patient education material is provided to patients.
